# Correction: Wang et al. Preparation of Electrospun Active Molecular Membrane and Atmospheric Free Radicals Capture. *Molecules* 2019, *24*, 3037

**DOI:** 10.3390/molecules31111813

**Published:** 2026-05-25

**Authors:** Guoying Wang, Ying Su, Jianglei Yu, Ruihong Li, Shangrong Ma, Xiuli Niu, Gaofeng Shi

**Affiliations:** 1 School of Petrochemical Engineering, Lanzhou University of Technology, Lanzhou 730050, China; su95ying@163.com (Y.S.); wx616534820@163.com (J.Y.); liruihong1006@126.com (R.L.); rren5856@gmail.com (S.M.); 2 Gansu Province Food Inspection Institute, Lanzhou 730050, China; liuyun420forever@163.com

In the original publication [1], there was a mistake in the subfigures for the SEM images of the spinning membrane of Figure 5.

Upon receiving the email, we immediately contacted the responsible authors of the paper and began a detailed review of the original data. We sincerely apologize for this error.

The correct SEM image appears below.

The authors state that the scientific conclusions are unaffected. This correction was approved by the Academic Editor. The original publication has also been updated.

The original publication has also been updated.

**Figure 5:**
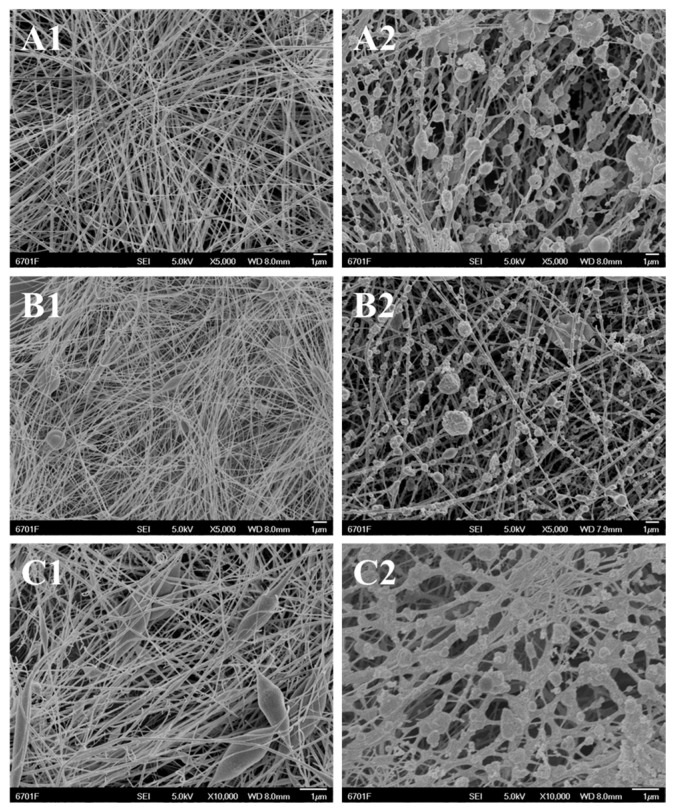
The SEM images of spinning membrane (**A1**: Quercetin; **B1**: Glycyrrhizic; **C1**: α-Mangostin); And after sampling (**A2**: Quercetin; **B2**: Glycyrrhizic; **C2**: α-Mangostin).
